# Incidence and timing of common adverse events in Lenvatinib-treated patients from the SELECT trial and their association with survival outcomes

**DOI:** 10.1007/s12020-017-1233-5

**Published:** 2017-02-03

**Authors:** Robert I. Haddad, Martin Schlumberger, Lori J. Wirth, Eric J. Sherman, Manisha H. Shah, Bruce Robinson, Corina E. Dutcus, Angela Teng, Andrew G. Gianoukakis, Steven I. Sherman

**Affiliations:** 1 Head and Neck Oncology Program, Dana Farber Cancer Institute, Brigham and Women’s Hospital, Boston, MA USA; 2 Department of Nuclear Medicine and Endocrine Oncology, Gustave Roussy and University Paris-Sud, Villejuif, France; 3 Department of Medicine, Massachusetts General Hospital, Boston, MA USA; 4 Department of Medicine, Memorial Sloan Kettering Cancer Center, New York, NY USA; 5 Department of Internal Medicine, The Ohio State University Comprehensive Cancer Center, Columbus, OH USA; 6 Kolling Institute of Medical Research, University of Sydney, New South Wales, Australia; 7 Clinical Development, Oncology Business Group, Eisai Inc., Woodcliff Lake, NJ USA; 8 Division of Endocrinology and Metabolism, Harbor-UCLA Medical Center, Torrance, CA USA; 9 Department of Endocrine Neoplasia and Hormonal Disorders, Division of Internal Medicine, The University of Texas MD Anderson Cancer Center, Houston, TX USA

**Keywords:** Lenvatinib, Adverse event, Dose reduction, Dose interruption

## Abstract

**Purpose:**

In the study of (E7080) lenvatinib in differentiated cancer of the thyroid, most patients experienced an adverse event. In this report, we examine common lenvatinib-emergent adverse events in this phase three, randomized, double-blind study.

**Methods:**

Adverse events were graded per Common Terminology Criteria for Adverse Events v4.0. 392 patients were enrolled (lenvatinib: 261, placebo: 131) and received lenvatinib 24 mg/day or placebo. The main outcome measures were: associations with progression-free survival and overall survival in exploratory univariate and multivariate analyses along with additional variables.

**Results:**

The most common any-grade adverse events (any grade; grade 3) in lenvatinib-treated patients included proteinuria (32%; 10%), diarrhea (67%; 9%), fatigue/asthenia/malaise (67%; 10%), rash (23%; 0.4%), and palmar-plantar erythrodysesthesia syndrome (33%; 3%). There were no grade 4 events for these adverse events. They generally occurred early (median time to first onset [weeks]: proteinuria [6.1], diarrhea [12.1], fatigue/asthenia/malaise [3.0], rash [7.3], and palmar-plantar erythrodysesthesia syndrome [5.9]), and were resolved primarily with dose modifications (median time to resolution [weeks]: proteinuria [8.8], diarrhea [18.1], fatigue/asthenia/malaise [16.3], rash [5.9], and palmar-plantar erythrodysesthesia syndrome [20.0]). Discontinuation due to these adverse events occurred in 2 (1%) patients with proteinuria and 4 (2%) with fatigue. Progression-free survival was not associated with any of the adverse events. Eastern Cooperative Oncology Group performance status (*P* = 0.001), follicular histology (*P* = 0.002), and diarrhea (*P* = 0.023) were associated with overall survival in multivariate analyses (median overall survival for patients with diarrhea: not reached; without: 17.1 months).

**Conclusions:**

In the study of (E7080) lenvatinib in differentiated cancer of the thyroid, the most common adverse events typically occurred early and were primarily managed with dose modifications. Overall survival was significantly associated with diarrhea.

## Introduction

Multikinase inhibitors, including those with antiangiogenic properties, have been increasingly positioned as the standard therapy either alone or in combination with other therapeutic agents for the treatment of multiple tumor types [1, 2]. Although the majority of antiangiogenic treatments are well tolerated, and toxicities are manageable with dose modifications, these agents are associated with distinct adverse events (AEs) because of their effect on tumors and the surrounding vasculature as well as on normal tissues [3]. Given the rapid expansion of these agents in clinical use, awareness of common toxicities and management is paramount, with the goal of providing optimal therapy for the patient.

Lenvatinib is an oral multikinase inhibitor of vascular endothelial growth factor receptor (VEGFR) 1–3, fibroblast growth factor receptor 1–4, platelet derived growth factor receptor α (PDGFRα), and RET and KIT proto-oncogenes [4–6]. Lenvatinib was recently approved for the treatment of radioiodine-refractory differentiated thyroid cancer (RR-DTC) in the United States, Europe, and Japan [7, 8], based on results from the pivotal phase 3 study of (E7080) lenvatinib in differentiated cancer of the thyroid (SELECT), where lenvatinib significantly prolonged progression-free survival (PFS) vs. placebo (median PFS: 18.3 vs. 3.6 months; hazard ratio [HR]: 0.21; 99% confidence interval [CI]: 0.14–0.31; *P* < 0.001) [9].

In SELECT, nearly all of the 392 patients enrolled experienced an AE. A large proportion of lenvatinib-treated patients required dose reduction (68%) or interruption (82%) due to treatment-emergent adverse events (TEAEs), but few required discontinuation (14%) of lenvatinib treatment due to TEAEs [9]. We had previously reported an analysis of lenvatinib-emergent hypertension—the most common AE in SELECT—its management, and correlations with efficacy from SELECT [10]. This current analysis examines the incidences, time course, and resolution of other clinically important and common lenvatinib-emergent AEs from SELECT—namely diarrhea, fatigue/asthenia/malaise, proteinuria, rash, and palmar-plantar erythrodysesthesia syndrome (PPES), and the relationship between these AEs and survival outcomes.

## Patients and methods

### Patients and study design

Full details of the SELECT methodology have been previously published [9]. Briefly, in this phase 3, randomized, double-blind, multicenter study, patients with RR-DTC and measurable disease per Response Evaluation Criteria in Solid tumors version 1.1 and independently reviewed radiologic evidence of disease progression within 13 months prior to study entry were enrolled. Patients could have received up to 1 prior VEGF-targeted therapy, and patients with proteinuria ≥1 g/24 h were excluded from the study. Eligible patients were stratified according to geographic region, age group, and prior VEGF-targeted treatment, and were randomized in a 2:1 ratio to receive oral doses of lenvatinib (24 mg once daily) or placebo in 28-day continuous cycles. Study treatment was administered until disease progression, development of unacceptable toxicities, or withdrawal of consent. A total of 392 patients enrolled (200 male and 192 female). 261 Patients were assigned to lenvatinib (125 male and 136 female) and 131 patients were assigned placebo (75 male and 56 female).

This study was conducted in accordance with the declaration of Helsinki and local laws. informed consent was obtained from all individual participants included in the study and the protocol was approved by all relevant institutional review bodies.

### Safety evaluation and treatment modifications

Safety assessments in SELECT included recording and monitoring all AEs and serious AEs, vital signs, clinical laboratory tests (hematology, clinical chemistry, urine values), and electrocardiograms. AEs were assessed based on the National Cancer Institute Common Terminology Criteria for Adverse Events, version 4.0 and coded according to the Medical Dictionary for Regulatory Activities version 16.0. In this analysis, all TEAEs were considered, regardless of clinical investigator-assessed relationship to study drug. Additionally, colitis, bowel movement irregularity, frequent bowel movements, functional gastrointestinal disorder, gastrointestinal disorder, and change in bowel habit were grouped together under diarrhea. Similarly, macule, papule, rash erythematous, rash generalized, rash macular, rash maculopapular, rash papular, and rash pruritic were included together under rash.

Lenvatinib dose interruptions for TEAEs of intolerable grade 2 or grade 3 were allowed until the events resolved to grade <1 or baseline, then sequential lenvatinib dose reductions (20 mg to 14 mg, then to 10 mg) were implemented. Patients discontinued treatment upon occurrence of a grade 4 TEAE, except for grade 4 laboratory abnormalities, which were treated as grade 3 events. Additionally, there was a specific monitoring plan for proteinuria, which was tested for at regular assessments during scheduled visits. During the randomization phase, patients with confirmed proteinuria based on a urine dipstick reading of ≥2 + were tested, at minimum, every 2 weeks until results were 1+ or negative for 3 consecutive months. Any subsequent proteinuria reading of ≥ 2 + by urine dipstick were assessed for AE grade and managed with dose modifications.

### Statistical analysis

The primary analysis data cutoff date for this study was 15 November 2013, and all enrolled patients who received any amount of lenvatinib or placebo in the randomization phase were included in the safety analysis. The time to onset of TEAEs were summarized using time-to-event analyses for median time to first onset, and descriptive statistics for cycle of first occurrence. Correlations between common TEAEs and efficacy endpoints (PFS and overall survival [OS]) were first analyzed using a Cox-proportional hazards model in univariate analyses, where factors with a *P* < 0.2 were further included in a multivariate model with additional variables (baseline Eastern Cooperative Oncology Group performance status [ECOG PS], previous VEGF-targeted therapy, baseline weight, age, region [Europe vs. Other], region [North America vs. Other], and histology [follicular vs. papillary thyroid cancer]). Sex was not considered a factor in this analysis.

## Results

### Frequency and time course of TEAEs

Of the 392 patients enrolled in SELECT, 261 received lenvatinib and 131 received placebo. All enrolled patients were included in the safety analysis. Incidence of treatment-related adverse events in SELECT was previously reported [9]. Overall, any-grade TEAEs (regardless of investigator-assessed relationship to study drug) occurred in 100% of lenvatinib-treated patients and 90% of placebo-treated patients. Grade 3 TEAEs occurred in 72% of lenvatinib-treated patients and 22% of placebo-treated patients, whereas grade 4 TEAEs occurred in 12% of lenvatinib-treated patients and 8% of placebo-treated patients.

Following hypertension, the most common TEAEs in lenvatinib-treated patients vs. placebo-treated patients included diarrhea (67% vs. 17%), fatigue/asthenia/malaise (67% vs. 35%), proteinuria (32% vs. 3%), rash (23% vs. 5%), and PPES (33% vs. 1%). The incidences of these common TEAEs, by grade 1–2 and grade 3, are described in Table 1. Importantly, there were no grade 4 events for any of these 5 TEAEs.

**Table 1:** Common lenvatinib-emergent adverse events

Lenvatinib-emergent adverse events	Grade 1–2n (%)	Grade 3n (%)	Median time to first onset, weeks (IQR)	Median time to last resolution, weeks (IQR)
Diarrhea^a^	152 (58)	23 (9)	12.1 (4.1, 23.7)	18.1 (2.3, 40.9)
Fatigue/asthenia/malaise	147 (56)	27 (10)	3.0 (1.1, 7.0)	16.3 (4.6, 36.6)
Proteinuria	58 (22)	26 (10)	6.1 (4.0, 15.6)	8.8 (4.0, 24.6)
Rash^b^	58 (22)	1 (0)	7.3 (2.9, 16.3)	5.9 (2.0, 18.6)
PPES	76 (29)	9 (3)	5.9 (3.1, 12.0)	20.0 (8.6, 32.1)

*IQR*, interquartile range, *PPES*, palmar-plantar erythrodysesthesia syndrome

^a^ Includes diarrhea, colitis, bowel movement irregularity, frequent bowel movements, functional gastrointestinal disorder, gastrointestinal disorder, and change in bowel habit

^b^ Includes macule, papule, rash, rash erythematous, rash generalized, rash macular, rash maculopapular, rash papular, and rash pruritic

**Fig. 1:**
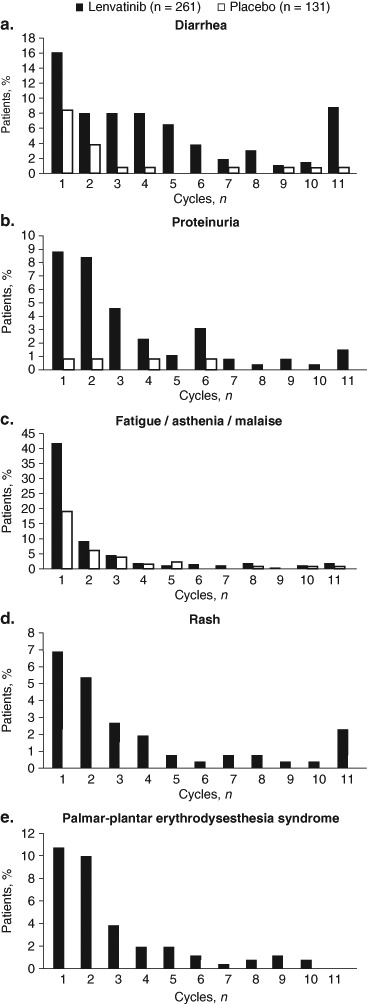
Cycle of first occurrence of adverse events (All Grades)

**Fig. 2:**
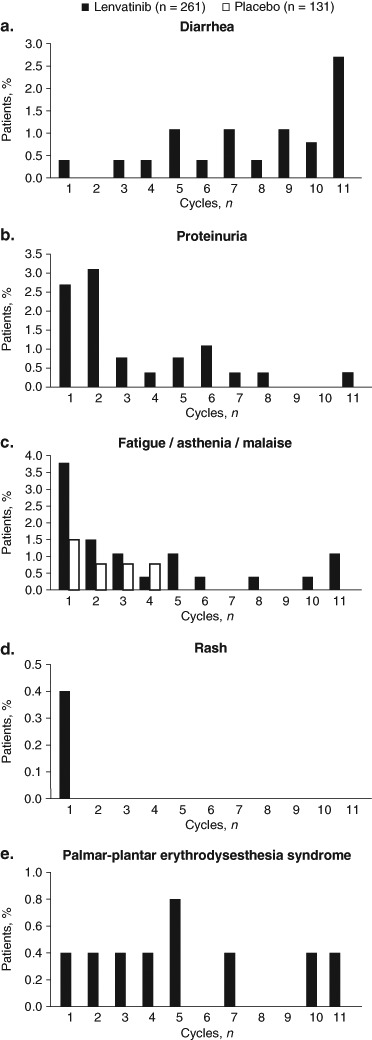
Cycle of first occurrence of grade 3 adverse events

The median time to first onset of any-grade TEAE was 12.1 weeks (interquartile range [IQR]: 4.1–23.7 weeks) for diarrhea, 3.0 weeks (IQR: 1.1–7.0 weeks) for fatigue/asthenia/malaise, 6.1 weeks (IQR: 4.0–15.6 weeks) for proteinuria, 7.3 weeks (IQR: 2.9–16.3 weeks) for rash, and 5.9 weeks (IQR: 3.1–12.0 weeks) for PPES in lenvatinib-treated patients. Generally, the frequencies of these TEAEs were higher in the earlier cycles of treatment and diminished over the course of treatment (Fig. 1). In cycle 1 of lenvatinib treatment, 16% of patients experienced diarrhea, 42% experienced fatigue/asthenia/malaise, 9% had proteinuria, 7% had rash, and 11% had PPES. In cycle 2 of lenvatinib treatment, 8% of patients had diarrhea, 9% had fatigue/asthenia/malaise, 8% had proteinuria, 5% had rash, and 10% had PPES. By cycle 6 of lenvatinib treatment, the frequencies of most TEAEs had reduced to ≤ 4%, with the exception of diarrhea at cycle 11 (9%). Overall, the frequencies of grade 3 events of these 5 TEAEs were low (< 4%) across treatment cycles Fig. 2.

### Management of TEAEs

Overall, TEAEs led to dose reductions or interruptions in 89% of the patients treated with lenvatinib in SELECT. In particular, these 5 common TEAEs were managed with supportive care, dose modifications, and, when necessary, concomitant medications (Table 2). Dose reductions and dose interruptions due to these TEAEs occurred in 1 to 13% and 0 to 18% of lenvatinib-treated patients, respectively, and only 6 patients discontinued lenvatinib treatment due to these TEAEs (4 patients with fatigue/asthenia/malaise and 2 patients with proteinuria). The median time to last resolution of each TEAE was 18.1 weeks (IQR: 2.3–40.9 weeks) for diarrhea, 16.3 weeks (IQR: 4.6–36.6 weeks) for fatigue/asthenia/malaise, 8.8 weeks (IQR: 4.0–24.6 weeks) for proteinuria, 5.9 weeks (IQR: 2.0–18.6 weeks) for rash, and 20.0 weeks (IQR: 8.6–32.1 weeks) for PPES in lenvatinib-treated patients.

**Table 2:** Management of common adverse events

Adverse Events, *n* (%)	Dose reduction		Dose interruption		Treatment discontinuation		Concomitant medication	
	LEN(*n* = 261)	PBO(*n* = 131)	LEN(*n* = 261)	PBO(*n* = 131)	LEN(*n* = 261)	PBO(*n* = 131)	LEN(*n* = 261)	PBO(*n* = 131)
Diarrhea^a^	27 (10)	0	46 (18)	0	0	0	111 (43)	8 (6)
Fatigue/asthenia/malaise	35 (13)	1 (0.8)	42 (16)	3 (2)	4 (2)	0	4 (2)	0
Proteinuria	28 (11)	0	42 (16)	0	2 (1)	0	2 (1)	0
Rash^b^	3 (1)	0	1 (0)	1 (1)	0	0	22 (8)	1 (1)
PPES	20 (8)	0	26 (10)	0	0	0	44 (17)	0

*LEN*, lenvatinib, *PBO*, placebo, *PPES*, palmar-plantar erythrodysesthesia syndrome

^a^ Includes diarrhea, colitis, bowel movement irregularity, frequent bowel movements, functional gastrointestinal disorder, gastrointestinal disorder, and change in bowel habit

^b^ Includes macule, papule, rash, rash erythematous, rash generalized, rash macular, rash maculopapular, rash papular, and rash pruritic

### TEAEs and associations with efficacy

Based on results of univariate analyses, out of the five examined TEAEs, only PPES (*P* = 0.08) was further included in a multivariate model for associations with PFS (Table 3). However, no significant association was found between lenvatinib-treated patients with PPES (*P* = 0.178) and PFS. The only significant association with PFS in the multivariate model was for baseline ECOG PS (favoring ECOG PS of 0 vs. ≥1; HR: 0.51; 95% CI: 0.34–0.76; *P* = 0.0008).

**Table 3:** Univariate and multivariate analyses for progression-free survival

Variables	Univariate analysis			Multivariate analysis		
	*HR*	95% CI	*P*-value	*HR*	95% CI	*P*-value
Fatigue/asthenia/malaise	1.16	0.75–1.78	0.509			
Diarrhea^a^	0.94	0.61–1.46	0.784			
PPES	1.49	0.95–2.33	0.084	1.35	0.87–2.08	0.178
Rash^b^	1.2	0.72–2.00	0.495			
Proteinuria	1.3	0.82–2.07	0.259			
Baseline ECOG PS (0 vs. ≥1)				0.51	0.34–0.76	0.0008
Prior VEGF therapy (0 vs. 1)				0.86	0.55–1.33	0.487
Baseline weight (kg)				0.99	0.98–1.00	0.078
Age (years)				1.02	1.00–1.04	0.084
Region (Europe vs. Other)				1.37	0.78–2.42	0.274
Region (North America vs. Other)				1.13	0.57–2.23	0.724
Histology (Follicular vs. papillary)				0.67	0.44–1.02	0.062

*CI*, confidence interval, *ECOG PS*, Eastern Cooperative Oncology Group performance status, *HR*, hazard ratio, *PPES*, palmar-plantar erythrodysesthesia syndrome, *VEGF*, vascular endothelial growth factor

^a^ Includes diarrhea, colitis, bowel movement irregularity, frequent bowel movements, functional gastrointestinal disorder, gastrointestinal disorder, and change in bowel habit

^b^ Includes macule, papule, rash, rash erythematous, rash generalized, rash macular, rash maculopapular, rash papular, and rash pruritic

For associations with OS, of the five examined TEAEs, only diarrhea (*P* = 0.012 in the univariate analysis) was further included as a variable in the multivariate model (Table 4). In the model, treatment-emergent diarrhea was found to be significantly associated with OS (yes vs. no; HR: 0.55; 95% CI: 0.33–0.92; *P* = 0.023), along with baseline ECOG PS (favoring ECOG PS of 0 vs. ≥1; HR: 0.44; 95% CI: 0.27–0.73; *P* = 0.001), and histology (favoring follicular vs. papillary thyroid cancer; HR: 0.36; 95% CI: 0.19–0.68; *P* = 0.002). The median OS for lenvatinib-treated patients who experienced diarrhea had not yet been reached at the time of data cutoff; however, for those patients who did not experience diarrhea, the median OS was 17.1 months (95% CI: 10.8–22.1).

**Table 4:** Univariate and multivariate analyses for overall survival

Variables	Univariate analysis			Multivariate analysis		
	*HR*	95% CI	*P*-value	*HR*	95% CI	*P*-value
Fatigue/asthenia/malaise	0.9	0.53–1.51	0.687			
Diarrhea^a^	0.5	0.29–0.86	0.012	0.55	0.33–0.92	0.023
PPES	0.72	0.40–1.30	0.276			
Rash^b^	0.77	0.39–1.52	0.449			
Proteinuria	1.22	0.71–2.10	0.477			
Baseline ECOG PS (0 vs. ≥ 1)				0.44	0.27–0.73	0.001
Prior VEGF therapy (0 vs. 1)				0.68	0.4–1.15	0.151
Baseline weight (kg)				0.99	0.98–1.00	0.174
Age (years)				1.01	0.98–1.03	0.64
Region (Europe vs. Other)				0.92	0.49–1.74	0.806
Region (North America vs. Other)				0.75	0.34–1.63	0.462
Histology (Follicular vs. papillary)				0.36	0.19–0.68	0.002

*CI*, confidence interval, *ECOG PS*, Eastern Cooperative Oncology Group performance status, *HR*, hazard ratio, *PPES*, palmar-plantar erythrodysesthesia syndrome, *VEGF*, vascular endothelial growth factor

^a^ Includes diarrhea, colitis, bowel movement irregularity, frequent bowel movements, functional gastrointestinal disorder, gastrointestinal disorder, and change in bowel habit

^b^ Includes macule, papule, rash, rash erythematous, rash generalized, rash macular, rash maculopapular, rash papular, and rash pruritic

## Discussion

In the phase 3 pivotal study of lenvatinib in patients with RR-DTC, lenvatinib met its primary endpoint and significantly improved PFS compared with placebo (18.3 and 3.6 months, respectively; HR: 0.21; 99% CI: 0.14–0.31; *P* < 0.001) [9]. The response rate with lenvatinib was also substantially improved (65% vs. 2% for placebo) [9]. At the time of the primary data cutoff (15 November 2013), the median duration of objective response had not been reached. In an updated analysis of response (data cutoff 31 August 2015), the median duration of objective response was 30 months (95% CI: 18.4–35.2) for lenvatinib and 14.7 months (95% CI 7.5–not evaluable) for placebo [11]. In an updated analysis of OS (updated data cutoff 15 June 2014) using a rank-preserving, structural-failure–time model to adjust for crossover, the HR for OS was 0.53; 95% CI: 0.34–0.82, with a nominal bootstrapped *P* = 0.0051. The median OS for lenvatinib still had not been reached, and was 19.1 months for placebo [12].

Similar to other established VEGF-targeted therapies for cancer, lenvatinib is associated with AEs that are distinct from cytotoxic treatment options [13, 14]. VEGF or VEGFR inhibitors are largely well-tolerated, and patients on these agents are less likely to discontinue treatment compared with cytotoxic therapies; however, with the chronic use of any agent, there may be the need for additional vigilance of side effects [15]. In addition, given the overall poor physical conditions of patients with advanced disease, clinicians should be prepared to monitor and manage AEs appropriately in order to improve or maintain patients’ quality of life [13]. Early assessment of AEs is particularly important so that early intervention can be implemented and patients can remain on treatment.

In the phase three SELECT study, toxicities were common, with almost all patients reporting a TEAE (lenvatinib, 100%; placebo, 90%). The most common TEAE was hypertension, which has been examined in a separate analysis [10]. In this analysis, the five common lenvatinib-emergent AEs profiled (diarrhea, fatigue/asthenia/malaise, proteinuria, rash, and PPES) typically occurred early—within the first 2 months of initiating therapy—and diminished over the course of treatment. The incidence of diarrhea was the highest during the first cycle, relatively stable between cycles two and five of lenvatinib treatment, and then decreased thereafter. Similarly, the frequency of fatigue/asthenia/malaise was highest during cycle one but was drastically reduced by cycle two onward. While clinicians should consider lenvatinib treatment for patients with progressive RR-DTC, it is important that preexisting conditions be adequately controlled before treatment. This is especially true for patients with preexisting hypertension, as this is the most common lenvatinib-emergent AE. However, in certain other settings, such as in patients with high risk for tracheoesophogeal fistula or bowel rupture, or in those already severely cachectic, any anti-angiogenic tyrosine kinase inhibitor therapy such as lenvatinib may be relatively contraindicated.

This general timing of AEs is similar to that found with other VEGF inhibitors [16], and may indicate that, for at least some of the AEs—for example, fatigue—symptoms may be self-limiting. Although it is unclear why the timing of these AEs follows such a pattern, it would be prudent to note that response to lenvatinib treatment is typically characterized by a rapid initial tumor size reduction (median change –25% by the first tumor assessment at 8 weeks), followed by slower but continuous shrinkage [17]. The magnitude of this reduction has been correlated to lenvatinib exposure during the first 8 weeks of treatment, which is also the period with the highest incidences of these profiled AEs. It is also possible that there is a reporting bias, and that reports of these AEs diminish over time as patients become accustomed to living with the symptoms. These findings altogether underscore the importance of timely and appropriate AE management by the treating physician, in order to maximize potential treatment benefit to the patients.

In previous studies of VEGF/R inhibitors, including sorafenib [18] and sunitinib [19], specific AEs were found to be predictive of superior survival outcome in patients with metastatic renal cell carcinoma. In SELECT, patients with lenvatinib-emergent hypertension may have a PFS advantage compared with patients treated with lenvatinib who did not experience hypertension [10]. In this current study, the occurrence of diarrhea, along with baseline ECOG PS and histology were identified as predictive factors for OS, whereas no examined AE in this analysis was predictive of PFS. It is important to note, however, that these analyses were exploratory in nature, and that there is no specific biologic hypothesis for an association between diarrhea and OS—diarrhea may be a pharmacodynamic marker, or the association may simply be one of chance.

In conclusion, these common TEAEs from SELECT—diarrhea, fatigue/asthenia/malaise, proteinuria, rash, and PPES―were manageable with dose modifications and supportive care, and very few patients discontinued lenvatinib treatment as a result of these events. The findings of this analysis thus provide insight regarding the need for proactive management of toxicities during lenvatinib administration, particularly after initiation of therapy, to maximize treatment benefit for the patient.
